# 

**Published:** 1877-09-20

**Authors:** 


					﻿VOL. VII.	SEPTEMBER 80, 1877	No. 9.
THE
SOUTHERN MEDICAL RECORD.
A MONTHLY JOURNAL OF PRACTICAL MEDICINE.
“ QUJCQUID PRJSCIPIES ESTO BREVIS.”
I £
W
Ph
i M
I H
I K
T
P5
P
I <1
O
i—i
A
' w
s
I pg
p
o
j
I O
I
p
<5
1
I p
I o
a?
; h-i
I W
: P
O
, oo
I S
* >—i
■ <1
P
Q
H
' Eh
£
QQ
S
Ph
‘ Ph
K
00
I s
Ph
□5 I
w
o
O
s I
g
□
3 I
M
a '
M
O
<32 j
> 1
d
hi
W l
> ■
31
*-H I
Q '
> I
b
1-1 I
H
00 |
00
O
t<
o
HH
JAS. P. HARRISON & CO., Printers.
,ATLANTA, GEORGIA.
TERMS: $2.00 per Annum in Advance—Club Rates, 5 Copies for $8.00.
				

